# Association between Screen Viewing Duration and Sleep Duration, Sleep Quality, and Excessive Daytime Sleepiness among Adolescents in Hong Kong

**DOI:** 10.3390/ijerph111111201

**Published:** 2014-10-28

**Authors:** Yim Wah Mak, Cynthia Sau Ting Wu, Donna Wing Shun Hui, Siu Ping Lam, Hei Yin Tse, Wing Yan Yu, Ho Ting Wong

**Affiliations:** 1 School of Nursing, the Hong Kong Polytechnic University, Hong Kong SAR, China; E-Mails: cynthia.wu@polyu.edu.hk (C.S.T.W.); htwong@polyu.edu.hk (H.T.W.); 2 Hong Kong Sanatorium & Hospital, Hong Kong SAR, China; E-Mails: donnawshui@gmail.com (D.W.S.H.); siaobing@gmail.com (S.P.L.); 3 Kwong Wah Hospital, Hong Kong SAR, China; E-Mail: heiyin98@gmail.com; 4 Pamela Youde Nethersole Eastern Hospital, Hong Kong SAR, China; E-Mail: wyy0728@yahoo.com.hk

**Keywords:** sleep quality, screen viewing, excessive daytime sleepiness, adolescents

## Abstract

Screen viewing is considered to have adverse impacts on the sleep of adolescents. Although there has been a considerable amount of research on the association between screen viewing and sleep, most studies have focused on specific types of screen viewing devices such as televisions and computers. The present study investigated the duration with which currently prevalent screen viewing devices (including televisions, personal computers, mobile phones, and portable video devices) are viewed in relation to sleep duration, sleep quality, and daytime sleepiness among Hong Kong adolescents (*N* = 762). Television and computer viewing remain prevalent, but were not correlated with sleep variables. Mobile phone viewing was correlated with all sleep variables, while portable video device viewing was shown to be correlated only with daytime sleepiness. The results demonstrated a trend of increase in the prevalence and types of screen viewing and their effects on the sleep patterns of adolescents.

## 1. Introduction

### 1.1. Sleep and Lifestyle

Sleep is crucial for the learning, memory processes, and school performance of adolescents [1]. (Adolescence is the period from the ages of 10 through to 19, and is the stage at which an individual’s lifestyle patterns are initiated and shaped [2,3]. This socially constructed definition was used in this study). Adequate sleep has been shown to boost the immune system, which helps to fight infections; thus, sleep may reduce a child’s risk of getting sick [4]. The psychological health of adolescents can be affected by sleep duration, with shorter sleep durations in adolescents having been linked to depression and an increase in suicide ideation [5]. According to the National Sleep Foundation [6], the recommended sleep duration for adolescents is 9 hours per night for optimum health and development. However, current research studies indicate that many adolescents get far less than this amount. In Hong Kong, a study conducted on 1629 adolescents aged 12 to 19 found the average daily sleep duration to be 7.3 h during weekdays [7]. In a survey study of 3478 Japanese high school students, the average sleep duration on weekdays was 6.3 h [8]. In South Korea, 1457 11th and 12th grade students reported getting only 5.4 h of sleep on school nights [9].

Lifestyle is a way of living based on identifiable patterns of behavior attributable to an individual’s personal characteristics and socioeconomic and environmental factors [10]. The versatile nature of individual behavioral patterns enables the early detection of health risks, so that behaviors can subsequently be modified to achieve optimum health. For adolescents, health is an important element in the path towards a productive and fruitful life, and lifestyle modification is regarded as a fundamental aspect of promoting health and preventing disease in adolescents [2]. Unhealthy behaviors that are established in adolescence can cause morbidity and mortality in adulthood [11]. It is therefore imperative to identify the health-risk behaviors of adolescents and intervene early in order to prevent long-term negative effects on health from keeping adolescents from achieving their full potential as competent members of society.

### 1.2. The Relationship between Lifestyle Pattern and Health

Among adolescents, sleep, physical activity, and screen viewing are common lifestyle patterns that are related to their health. These activities can also occupy the majority of their time—more than 14 h daily [12,13]. It has been well-established that adequate sleep and physical activity are essential for adolescent growth; yet the majority of adolescents in many developed countries reported getting inadequate amounts of sleep and engaging in less physical activity than the recommended level [14,15]. Moreover, adolescents live in an era of continued advancements in technology, resulting in a trend of increase in the ownership and use of screen viewing devices, including televisions, computers, mobile phones, and various types of portable devices [16]. Inadequate sleep and physical activity, compounded by increasing amounts of time spent viewing the abovementioned devices, may ultimately cause the health of adolescents to suffer.

### 1.3. Sleep Outcome

According to the National Sleep Foundation [6], sleep duration is not the only indicator of sleep. Sleep quality and excessive daytime sleepiness are significant indicators of sleep outcome. Sleep quality refers to continuous sleep without any interruption [6]. It can also be characterized by the occurrence of certain conditions such as the early onset of sleep, fewer interruptions, and fewer early awakenings. Good sleep quality is associated with a wide range of positive outcomes such as better health, greater well-being, and better psychological functioning among adolescents [17]. Inadequate or disrupted sleep can directly result in excessive daytime sleepiness [18,19]. Adolescents with daytime sleepiness are likely to experience reduced alertness, compromised daytime functioning, and impaired mood [18,20,21].

However, adolescents worldwide have been shown to experience poor sleep quality. Chung and Cheung [5] reported that 19.1% of Hong Kong adolescents experience difficulties in falling asleep, wake up during the night, or wake up too early in the morning, while 20% of students stated that they needed more than 30 min to fall asleep. Excessive daytime sleepiness was also experienced by 41.9% of the adolescents. According to a Japanese study of 102,451 high school students, the prevalence of difficulty in falling asleep, difficulty maintaining sleep, and early morning awakening was 14.8%, 11.3%, and 5.5%, respectively; 23.5% of adolescents also reported having one or more symptoms of insomnia [22]. Gibson *et al.* [23] reported that 41.2% of 3235 Canadian high school students experience excessive daytime sleepiness. In the U.S., 12.4% of 5118 ninth-grade students were found to have experienced insomnia almost every day of the preceding month [24], while 26.8% of 11 to 17 year olds experienced difficulty in initiating or maintaining sleep [25]. Evidently, poor sleep quality for adolescents is a global health concern of startling proportions.

### 1.4. Screen Viewing

Screen viewing has become a crucial activity in the everyday life of adolescents. According to the American Academy of Pediatrics [26], the recommended daily screen viewing time should be no more than one to two hours per day. The increasing prevalence of screen-based activities is known to be associated with other lifestyle and health-related problems among adolescents. For example, an increase in the duration of watching television and playing with computers was associated with a decrease in the likelihood of adolescents adopting healthy behaviors such as appreciating life, taking responsibility for one’s health, and forming social support networks [27].

Numerous research studies have illustrated that the individual viewing of televisions and computers has exceeded the recommended level in duration. In Hong Kong, the average amount of time that adolescents spent daily watching television increased from 2 h and 50 min to 3 h and 27 min from 1995 to 2008 [28]. In addition, one study found that the amount of time that Hong Kong adolescents spent daily on using computers was 2 h and 30 min [29]. In comparison, in the U.S., the average amount of time per day that adolescents spent on using computers increased from 58 min in 1999 to 2 h and 17 min in 2009—an increase of 136% within 10 years [30].

Apart from televisions and computers, mobile phones and portable video devices are multi-functional gadgets that adolescents can use anytime and anywhere. In the U.S., 66% of adolescents own a mobile phone and spend an average of 1 h and 35 min on texting, 17 min on playing games, and 15 min on watching television [30]. In Hong Kong, 87% of adolescents own a mobile phone, the highest percentage in Asia [31]. Popular portable video devices include iTouch, NDS, and PSP. In contrast to iTouch, which is a multifunctional device, playing video games is the main function of NDS and PSP [32,33]. In the U.S. 55% of adolescents own a portable video gaming device, compared to 40% of their Hong Kong counterparts [31,34]. The average amount of time that American adolescents spent using portable video gaming devices increased from 17 min in 2004 to 38 min in 2009 [30]. This indicates that the prevalence of screen viewing is likely to increase with the emergence of new types of screen viewing devices. As a result, lifestyle patterns such as sleep may be compromised, which in turn may have harmful effects on adolescent health.

### 1.5. Research Gap

Previous studies examining the association between screen viewing and sleep found that the prolonged watching of television and use of computers is associated with inadequate sleep and delayed bedtimes [35,36,37,38]. Punamäki *et al.* [39] emphasized that the association between computer usage and inadequate and irregular sleep is more significant for those in early adolescence than for older adolescents. Lastly, the excessive watching of television and use of computers has also been associated with excessive daytime sleepiness in adolescents [40,41].

Gaps have been identified in what is known about this topic. Although the association between television and computer viewing and sleep has been established, the impact of mobile phones and portable video devices, as well as of the proliferating varieties of screen viewing devices, on sleep duration, sleep quality, and daytime sleepiness has rarely been explored. In addition, since most studies examined the impact of individual types of screen viewing devices, studies on the association between total screen viewing duration on sleep duration, sleep quality, and daytime sleepiness are limited. Finally, although the association between screen viewing and sleep has been extensively covered in overseas studies, the effects of screen viewing on Hong Kong adolescents have not been well established. In addressing these gaps in knowledge, the present study will provide information on screen viewing, sleep duration, sleep quality, and daytime sleepiness among adolescents. It will also provide insights on the potential health consequences of cumulative exposure to screen viewing among Hong Kong adolescents. Due to the large scale of the present study, this manuscript will focus on the association between an increase in the duration of screen viewing and sleep.

### 1.6. Aim and Objectives

The aim of the present study is to explore the impact of screen viewing on sleep duration, sleep quality, and daytime sleepiness among Hong Kong adolescents. The objectives are (1) to describe the screen viewing duration, sleep duration, sleep quality, and excessive daytime sleepiness of Hong Kong adolescents; and (2) to examine the association between screen viewing duration and: (i) sleep duration, (ii) sleep quality; and (iii) excessive daytime sleepiness in Hong Kong adolescents.

## 2. Methods

### 2.1. Participants

The participants were recruited from five secondary schools through a two-stage process of random sampling. The Sham Shui Po district was randomly selected from 18 districts in the first stage; five secondary schools from 39 secondary schools in Sham Shui Po were then selected in the second stage. A total of 817 participants were approached from one class in each form, 792 of whom returned completed questionnaires, yielding a response rate of 97%. Thirty questionnaires were excluded on the basis that more than 20% of the questions had not been answered, or that the questions in the entire screen viewing domain had been ignored. The final sample consisted of 762 participants.

The mean age of the participants was 15.27 years (SD = 1.70, ranging from 12 to 20 years), and 57.6% of the participants were boys (*n* = 434) and 42.4% were girls (*n* = 319). The participants were evenly distributed in terms of grade level in school. Most of the participants had a monthly household income of HK$4000 to HK$14,999, which is consistent with the monthly household income distribution in Hong Kong. A majority of adolescents reported that their health was good or very good (62.8%), but 55.2% reported having experienced somatic pain in the preceding one-month period.

### 2.2. Research Design

A cross-sectional survey was used to examine the information on the participants’ screen viewing duration, sleep duration, sleep quality, and daytime sleepiness, as well as their demographic characteristics.

### 2.3. Procedure and Ethical Considerations

This study was conducted from May to June 2011. The purpose of the study was explained to the principals of the target schools. In loco parentis, permission was obtained from the principals. With the approval of the selected schools, school teachers distributed information sheets containing the purpose and objectives of the study, along with reply slips, to the students to give to their parents. A passive consent method was used and the reply slip was only required if parents refused to let their children participate in the study. A higher response rate can be achieved with a passive consent method [42], as those who did not return the reply slip indicating their refusal were assumed to have given consent for their children to participate in the study. Students who were eligible to participate were given the questionnaires in class, along with instructions from their teachers. Parents were informed that the survey was anonymous, and the students were told that they were allowed to withdraw at any time without penalty. Researchers then collected the completed questionnaires. The study was conducted in accordance with the Declaration of Helsinki, and the protocol was approved by the Human Research Ethics Approval Committee of Hong Kong Polytechnic University on 20 May 2011.

### 2.4. Measures

Sleep duration, sleep quality, and daytime sleepiness. Modified and extracted items from the Pittsburgh Sleep Quality Index (PSQI) were used to examine sleep-wake patterns. These consisted of six items on sleep time, wakeup time, and sleep duration on weekdays and weekends [43]. The participants were asked to indicate what time they usually go to bed and wake up on an average weekday (Monday to Friday) and on an average day of the weekend (Saturday to Sunday). Sample items include: (1) When have you usually gone to bed on a weekday (Mon-Fri) night (clock time)? (2) When have you usually gotten out of bed on a weekday (Mon-Fri) morning (clock time)? (3) How many hours of actual sleep do you get at night (starting from when you fell asleep) (hours and minutes)? The average daily sleep duration was calculated by the sum of the sleep duration on weekdays and weekends divided by seven.

The Chinese version of the Sleep Quality Index (SQI), an eight-item questionnaire, was used to examine the disturbances to the adolescents’ sleep in the preceding one-month period. The quality of the adolescents’ sleep was assessed by the occurrence of difficulty in falling asleep, waking up during the night, waking up too early in the morning, disturbed sleep at night, and insomnia. Responses were weighted as 0, 1, or 2, with 2 representing the highest severity [7]. The total score ranges from 0 to 16, with 0 to 1 representing good sleep quality, 2 to 8 indicating occasional sleep difficulties, and 9 or above indicating poor sleep quality [16].

The Chinese version of the Epworth Sleepiness Scale (ESS) was used to assess excessive daytime sleepiness in adolescents. It consists of a four-point scale to rate the likelihood of dozing in eight daily life situations. The situations include sitting and reading, watching television, sitting and talking to someone, sitting quietly after a lunch without alcohol, and so on [7]. The total score ranges from 0 to 24, with a total score of more than 10 indicating excessive daytime sleepiness [44].

Screen viewing. The participants were asked to report the exact amount of time that they spent viewing televisions, computers, mobile phones, and portable video devices on weekdays (Monday to Friday) and over the weekends (Saturday and Sunday). They were also asked a follow-up question on their self-reported total screen viewing duration on weekdays and weekends. The daily screen viewing duration for each type of device was calculated by the sum of the screen viewing duration (each type) during weekdays and weekends divided by seven. A separate calculation of total screen viewing duration was made by the researchers by adding up the daily screen viewing durations for each type of screen viewing device. The calculated total screen viewing duration differed from the self-reported total screen viewing duration. As some of the respondents may have had the habit of using multiple devices at the same time, they may have had difficulty inaccurately estimating the amount of time that they spent viewing each device. The calculated total screen viewing duration of some of the respondents exceeded 24 h, which is certainly not possible. Hence, the calculated daily total screen viewing duration in this study was used only for reference, and discrepancies in the self-reported and calculated daily total screen viewing duration for televisions, computers, mobile phones, and portable video devices indicate the use of multiple screens. Although self-reported screen viewing time has its own limitations, this measure is not infrequently used in academic research [45,46,47].

Socio-demographic variables. This section consists of data on the participants’ gender, age, level of education, monthly household income, and parental education levels.

All of the measures used in the study were psychometrically reliable and valid. The Cronbach’s alpha for the PSQI, SQI, and ESS were 0.71 [43], 0.74 [48], and 0.85 [49] respectively, indicating moderate to strong internal consistency. According to Tsai *et al.*, the test-retest reliability coefficients of the PSQI were 0.85 (*p* < 0.001), while that of the ESS was 0.74 (*p* = 0.001) [43]. The intra-class coefficients of the screen viewing variables, SQI, and ESS, were greater than 0.7, which is satisfactory [50]. Validity was evaluated in terms of content validity by three panel members who are experts in sleep science, lifestyle and health behaviors, and quality of life. The constructs of screen viewing, sleep duration, SQI, ESS, and the overall Scale-Content Validity Index (S-CVI) were greater than 0.94, demonstrating that the questionnaire had good content validity [50].

### 2.5. Data Analysis

All statistical analyses were performed using the Predictive Analytics Software Statistics (PASW) for Windows, version 18.0. Descriptive statistics for continuous data were made using the mean, median, standard deviation (SD), and interquartile range (IQR). Categorical data were described using frequencies and percentages. The screen viewing duration, sleep duration, sleep quality, daytime sleepiness, and socio-demographic characteristics of the participants were descriptively analyzed. Due to the skewness of the data, a Spearman’s Rho Correlation test was used to examine the association between screen viewing duration and (1) sleep duration; (2) sleep quality; and (3) daytime sleepiness. Finally, multivariate linear regression models were developed using the stepwise variable selection method for the above three sleep outcomes as dependent variables. Before the regression analysis was conducted, a bivariate analysis was done between the socio-demographic variables in Table 1 and the sleep outcomes, and only socio-demographic variables and types of screen viewing variables significantly related to the sleep outcomes were included in the regression analysis. The level of statistical significance was set at 0.05.

**Table 1:** Descriptive Statistics of the Participants (*n* = 762).

Variables *		*n* (%)	
Gender (*n* = 753)	Male	434 (57.6)	
	Female	319 (42.4)	
Age (*n* = 746)	12	38 (5.1)	
	13	93 (12.5)	
	14	117 (15.7)	
	15	146 (19.6)	
	16	175 (23.5)	
	17	104 (13.9)	
	18	57 (7.6)	
	19	13 (1.7)	
	20	3 (0.4)	
Grade in school (*n* = 758) **	Form 1	159 (21.0)	
	Form 2	165 (21.8)	
	Form 3	172 (22.7)	
	Form 4	128 (16.9)	
	Form 5	134 (17.7)	
Monthly household income **^,# #^ (*n* = 708)	Below HK$4000	62 (8.8)	
	HK$4000–14,999	432 (61.1)	
	HK$15,000 +	214 (30.2)	
Parental education level: Father (*n* = 607)	Primary or lower	117 (19.2)	
	Secondary	450 (74.2)	
	Tertiary	40 (6.6)	
Parental education level: Mother (*n* = 611)	Primary or lower	144 (23.6)	
	Secondary	436 (71.4)	
	Tertiary	31 (5.1)	
Perceived general health condition in the past 1 month (*n* = 727)	Very good	134 (18.4)	
	Good	323 (44.4)	
	Fair	226 (31.1)	
	Poor	44 (6.1)	
Experienced somatic pain in the past 1 month (*n* = 667)	Yes	348 (55.2)	
	No	283 (44.8)	
Other disease that required medical follow up (*n* = 754)	Yes	41 (5.4)	
	No	713 (94.6)	

Notes: * The total for all variables may not add up to 762 due to missing data. The percentage of missing data is as high as 7%; ** Forms 1 to 5 are equivalent to the 7th to 11th grades in the U.S. education system; *** Monthly household income is categorized based on the categories in the Census and Statistics Department’s Household Income Distribution in Hong Kong [51]; ^#^ U.S.$1 is rated at HK$7.8; ^##^ According to the 2006 Census, the percentages of those in the income groups of below HK$4000, HK$4000–$14,999, and HK$ ≥ 15,000 are 11.7, 56.9, and 31.5, respectively.

## 3. Results

Descriptive statistics on the participants are presented in Table 1. The adolescents reported that they got an average of 7.74 h of sleep per night (see Table 2); however, 55.6% (*n* = 414) of them reported getting less than 8 hours of sleep per night. The mean ESS total score was 6.96 (SD = 3.99), with 17.6% (*n* = 133) of the adolescents having an ESS total score of higher than 10. The mean SQI total score was 4.11 (SD = 2.88).

**Table 2:** Sleep Duration of the Participants.

Age Group	Time (Hours/Night) *			
	<8 h	≥8 h	Mean ± SD	Median (Interquartile Range)
12–13 (*n* = 130)	53 (40.8)	77 (59.2)	8.23 ± 2.50	8.23 (7.0–9.0)
14–15 (*n* = 254)	137 (53.9)	117 (46.1)	7.80 ± 1.61	7.86 (7.0–8.4)
16–17 (*n* = 275)	176 (64.0)	99 (36.0)	7.54 ± 1.31	7.57 (6.9–8.2)
≥18 (*n* = 72)	39 (54.2)	33 (45.8)	7.83 ± 1.18	7.82 (7.0–8.6)
All (*n* = 745)	414 (55.6)	331 (44.4)	7.74 ± 1.46	7.82 (7.0–8.5)

Note: * According to the National Sleep Foundation [52], sleep duration for adolescents of less than 8 h per day reflects insufficient sleep.

The adolescents spent the most time on computers (3 h and 26 min), followed by watching television (2 h and 50 min), then using mobile phones (2 h and 18 min and portable video devices (1 h and 3 min) (see Table 3). 99.1% (*n* = 742) of the adolescents reported using a computer, 97.6% (*n* = 731) reported watching television, 85.8% (*n* = 647) reported using a mobile phone, and 49.6% (*n* = 373) reported using portable video devices. On average, the adolescents spent a total self-reported 5 h and 54 min per day on using screen viewing devices, with most adolescents reporting a high level of screen use (23.7%).

**Table 3:** Prevalence and Duration of Screen Use by the Participants.

Type of Screen Viewing Device	Count *n* (%)		Time (Hours/d)		
			*n* (% )	Median (Interquartile Range)	Mean ± SD
	Yes *	No			
Television (*n* = 749)	731 (97.6)	18 (2.4)		2.29 (1.29–3.64)	2.84 ± 2.21
Low (<1h)			95 (12.7)		
Medium (≥1–2 h)			231 (30.8)		
High (>2–3 h)			171 (22.8)		
Very high (>3–4 h)			103 (13.8)		
Extremely high (>4 h)			149 (19.9)		
Computer (*n* = 749)	742 (99.1)	7 (0.9)		3.00 (1.64–4.57)	3.44 ± 2.36
Low (<2h)			231 (30.8)		
Medium (≥2–3 h)			156 (20.8)		
High (>3–4 h)			123 (16.4)		
Very high (>4 h)			239 (31.9)		
Mobile Phone (*n* = 754)	647 (85.8)	107 (14.2)		1.00 (0.29–2.39)	2.31 ± 3.74
Low (<0.5 h)			227 (30.1)		
Medium (≥0.5–1 h)			204 (27.1)		
High (>1–2 h)			109 (14.5)		
Very high (>2 h)			214 (28.4)		
Portable video device (*n* = 752)	373 (49.6)	379 (50.4)		0.00 (0–1.17)	1.05 ± 2.30
None (0 h)			379 (50.4)		
Low (>0–1 h)			176 (23.4)		
High (>1 h)			197 (26.2)		

Note: * Indicates participants who have used that particular screen viewing device.

Table 4 shows the results of the Spearman’s Rho Correlation test in which the screen viewing and sleep variables were tested as continuous data and the correlation coefficients were listed. There was no significant correlation between daily self-reported television viewing duration and daily average sleep duration, sleep quality, and daytime sleepiness. Daily self-reported computer viewing duration was significantly correlated with only daytime sleepiness (*r* = 0.101, *p* < 0.01). Daily self-reported mobile phone viewing duration was significantly correlated with daily average sleep duration (*r*= −0.074, *p* < 0.05), sleep quality (*r* = 0.085, *p* < 0.05), and daytime sleepiness (*r* = 0.145, *p* < 0.001). Daily self-reported portable video device viewing duration was found to be correlated with daily average sleep duration alone (*r* = −0.076, *p* < 0.05). The total self-reported screen viewing duration was correlated with daytime sleepiness (*r* = 0.087, *p* < 0.05), while the total calculated screen viewing duration was correlated with sleep quality (*r* = 0.126, *p* < 0.001) and daytime sleepiness (*r* = 0.137, *p* < 0.001).

**Table 4:** Association between types of screen viewing and sleep duration, sleep quality, and daytime sleepiness.

Screen Viewing Variables	Daily Average Sleep Duration (Hours)	Total SQI Score	Total ESS Score
Daily self-reported television viewing duration (Hours)	0.050	0.047	0.038
Daily self-reported computer viewing duration (Hours)	−0.020	0.051	0.101 **
Daily self-reported mobile phone viewing duration (Hours)	−0.074 *	0.085 *	0.145 ***
Daily self-reported portable video device viewing duration (Hours)	−0.076 *	0.069	0.049
Total self-reported screen viewing duration (Hours)	−0.005	0.018	0.087 *
Total calculated screen viewing duration (Hours)	−0.038	0.126 ***	0.137 ***

Notes: * p < 0.05, ** *p* < 0.01, *** *p* < 0.001.

Table 5 is the result of the regression analysis. For daily average sleep duration, no screen viewing predictor was found after controlling for the factor of grade in school. However, the factor of self-reported mobile phone viewing duration was found to be a significant predictor of the total SQI Score (B: 0.101; 95% CI: 0.033–0.170; *p* < 0.01) and the total ESS Score (B: 0.117; 95% CI: 0.015–0.219; *p* < 0.05) after controlling for socio-demographic variables. The positive regression coefficients suggest that mobile phone viewing duration increases the risk of poor sleep quality and excessive daytime sleepiness.

**Table 5:** Screen Viewing Predictors of Sleep Outcomes.

Model Number ^†^	Dependent Variable	Screen Viewing Predictors	B	95%CI	*p*-value
1 ^#^	Daily Average Sleep Duration	-	-	-	-
2 ^##^	Total SQI Score	Self-reported mobile phone viewing duration	0.101	(0.033,0.170)	0.004 **
3 ^###^	Total ESS Score	Self-reported mobile phone viewing duration	0.117	(0.015,0.219)	0.024 *

Notes: Controlled socio-demographic variables: ^#^ Grade in school; ^##^ Perceived general health condition, Experienced somatic pain; ^###^ Grade in school, Perceived general health condition; ^†^ Total calculated screen viewing duration was not considered. * p < 0.05, ** *p* < 0.01, *** *p* < 0.001.

## 4. Discussion

### 4.1. Sleep Outcomes

Adolescents in Hong Kong reported an average sleep duration of 7.74 h, suggesting that they are getting an inadequate amount of sleep, given the recommendation by the U.S. Department of Health and Human Services of 10 h for school-aged children and adolescents [53]. It is also less than the recommended sleep duration for adolescents suggested by Carskadon *et al.* [54] for optimal daytime alertness. Comparing studies from overseas, the sleep duration reported in this study is similar to that in Canada [23], but less than that in mainland China [55], and more than that in Japan [8] and South Korea [9].

Hong Kong adolescents were shown to experience occasional sleep difficulties as well. Three of the most prevalent sleep problems experienced by adolescents were morning tiredness, taking more than 30 min to fall asleep, and waking too early in the morning, with prevalence rates of 34.1%, 13.6%, and 12.7%, respectively. This finding is similar to that of a local study conducted among adolescents in 2003 [7]. Although different instruments or criteria were used to measure sleep quality in adolescents in other countries, similar sleep complaints were mentioned in those studies. Compared to a study conducted in the U.S. [52], the prevalence of morning tiredness, a longer time needed to fall asleep, and disturbed night sleep were lower among Hong Kong adolescents. With regard to insomnia, the prevalence is lower in Hong Kong than in Japan, China, and the U.S., where the prevalence rates were 23.5%, 16.1%, and 25%, respectively [22,55,56].

Interestingly, excessive daytime sleepiness was not common among Hong Kong adolescents, with only 17.6% of adolescents reporting an ESS score of greater than 10. This finding is much lower than that in Canada, where a prevalence rate of 41.5% was reported among 3235 adolescents aged between 14 to 18 [23]. The discrepancy in the prevalence of excessive daytime sleepiness among adolescents may be due to their different racial and social backgrounds, as well as to the different versions of the ESS that were used.

### 4.2. Screen Viewing

Our finding confirmed that the prevalence of television and computer viewing has reached a near saturation level of 97.6% and 99.1%, respectively. The prevalence of computer viewing is in accord with the findings in other developed countries such as South Korea, Singapore, Japan, and the U.S. [57]. Compared with the finding from Synovate [31], the prevalence of mobile phone viewing was approximately the same at 86%; however, there was a nearly 10% increase in the prevalence of portable video device viewing. The prevalence of mobile phone viewing among Hong Kong adolescents was lower than that in Singapore and Japan, but higher than that in Korea, Taiwan, and the U.S. [57]. Although nearly 50% of Hong Kong adolescents own a portable video device, the prevalence of portable video device viewing was still the lowest among the four types of screen viewing devices examined in our study.

Computer viewing duration was the highest, followed by television, mobile phone, and portable video device viewing. The average computer viewing duration was almost 3 h per day, which represents an increase of 30 min within 10 years [29]. The average television viewing duration was 2.29 h per day, which is more than the recommended level of no more than 2 h a day [58]. However, our study indicated that almost 60% of adolescents failed to meet the recommended level. Comparing studies conducted in Canada [59] and the U.S. [30], the average computer viewing duration is highest among Hong Kong adolescents, while the average television viewing duration among Hong Kong adolescents is similar to that of adolescents in Canada, but less than that of adolescents in the U.S.

Regarding mobile phone and portable video device viewing among Hong Kong adolescents, the average daily mobile phone viewing duration of 1 hour is less than that among U.S. adolescents at 2.11 h [30]. As the distribution of portable video device viewing duration was highly skewed, the median duration of portable video device viewing was shown to be zero. It is difficult to draw comparisons between our finding on portable video device viewing duration with that of other overseas studies, as the concept of “video game playing” comprised the use of televisions, computers, and portable video devices [51,57].

### 4.3. Association Between Screen Viewing and Sleep Outcomes: Television 

This study is the first study in Hong Kong to examine the association between screen viewing duration and sleep duration, sleep quality, and daytime sleepiness among secondary school students. It illustrates that certain types of screen viewing are correlated with shorter sleep duration, greater sleep disturbances, and daytime sleepiness. Among the four screen viewing media, television viewing was found to have no correlation with any sleep variables, which is inconsistent with the findings of most international studies. Li *et al.* [60] examined the risk factors associated with short sleep durations among school-aged children in China. After adjusting for demographic and socioeconomic variables, they found that short sleep duration was associated with more than two hours of television viewing during weekdays. A study of 2,546 adolescents in Belgium concluded that adolescents who were more frequent viewers of television spent less time in bed on weekdays, and reported higher overall levels of tiredness [38]. Similarly, the same association between increased television viewing and shorter sleep durations was found among Spanish adolescents, who were more likely to be tired in the morning than in the other time of a day after controlling for age [36]. A longitudinal study in the U.S. investigated the association between television viewing and sleep problems during adolescence. It was found that adolescents who watched three hours or more of television per day during adolescence had more frequent sleep problems by early adulthood. Their results remained significant even after controlling for socio-demographic variables [61]. These international studies have all controlled for confounding variables such as age, gender, and parental education levels. Since the Spearman’s rho correlation did not allow for the controlling of confounding variables, this may be the major reason for the inconsistency between these studies and our results on the association between television viewing duration and sleep.

### 4.4. Association between Screen Viewing and Sleep Outcomes: Computer

Computer viewing duration was found to not be associated with sleep duration and sleep quality, but to be correlated with daytime sleepiness. The lack of correlation may again be due to the lack of control of confounding factors. As for the correlation between computer viewing duration and daytime sleepiness, this is consistent with the findings of some overseas studies. A study conducted in Finland on 7292 adolescents showed that intensive computer usage was negatively associated with sleeping habits, which in turn was associated with increased daytime sleepiness [39]. The increase in daytime sleepiness associated with computer viewing may be caused by the increased excitement and psychological arousal triggered by gaming at night.

### 4.5. Association between Screen Viewing and Sleep Outcomes: Mobile Phone

Mobile phone viewing duration was found to be correlated with sleep duration, sleep quality, and daytime sleepiness. This is consistent with a study on junior high school students in Japan regarding mobile phone usage and sleep patterns, where it was found that mobile phone usage of over twenty minutes was enough to delay bedtimes and wake-up times and to result in a reduction in sleep duration [62]. Punamäki *et al.* [39] also demonstrated that mobile phone usage was intensive among girls, which was associated with daytime sleepiness. It was suggested that such intensive mobile phone usage may in large part be due to the girls’ stage of development, when close friendships and the sharing of secrets and experiences are important. The girls may therefore feel the need to communicate constantly through mobile phones, which may reduce their sleep duration and in turn increase their daytime sleepiness [39]. However, Yen *et al.* [63] did not find an association between mobile phone viewing and short sleep duration in adolescents in Taiwan, as there may be differences among individuals.

The association between mobile phone viewing duration and sleep may also be due to an increase in the current accessibility and availability of mobile phones, which increases the chance of adolescents using mobile phones anytime and anywhere [37]. Moreover, that mobile phone viewing duration was the only significant screen viewing predictor of the total SQI and ESS scores also indicates the importance of mobile phones. The popularity of smart mobile phones suggests that they may ultimately dominate other traditional digital devices, such as television, in the area of the effects of screen viewing on sleep outcomes. In addition, mobile phone viewing is seen as an unstructured and unmonitored activity; adolescents can therefore continue viewing without any parental supervision, which may alter their sleep patterns, decrease their sleep duration, and increase their daytime sleepiness [60,63]. In terms of the physiological and psychological aspects of mobile phone viewing, the contents of exposure may cause nervous arousal, as well as neck and shoulder pains that may reduce sleep quality [39]. Studies have shown that exposure to bright light may affect the sleep-wake cycle through the suppression of the nocturnal salivary secretion of melatonin, which contributes to such sleep disturbances as repeatedly awakening at night [39,64]. Phone calls and messages received at night may also activate areas of the brain that are responsible for sleep and calming down [65].

### 4.6. Association between Screen Viewing and Sleep Outcomes: Portable Video Devices

It was found that portable video device viewing is negatively correlated only with sleep duration. As no studies have been conducted on portable video device viewing and sleep, it can only be suggested that the negative association may be caused by the unstructured nature of gaming through portable video devices, with adolescents able to engage in gaming until late at night, which delays their bedtime and shortens their sleep duration. Gaming can also induce psychological effects that may disrupt sleep and reduce sleep duration.

### 4.7. Multiple Screen Viewing

According to Rideout *et al.* [30], the percentage of adolescents using multiple screen viewing devices is perhaps as high as 29%. Multiple screen use was also found in our study, as substantiated by the discrepancy between the total self-reported screen viewing duration (5.29 h per day) and the total calculated screen viewing duration (7.64 h per day). As most studies have only reported on specific types of screen viewing devices, it can only be suggested that the discrepancy is largely due to the underreporting of multiple screen use.

## 5. Study Strengths and Limitations

The strength of this study is that it is a preliminary study that provides information to understand the prevalence and duration of usage of a comprehensive set of screen viewing devices. It also offers a current picture of the sleep quality of healthy adolescents in Hong Kong. Its relatively simple design made it possible to recruit a large number of subjects in a school-based study.

Limitations include the cross-sectional design, which could not determine the relationship of cause-and-effect between screen viewing duration and sleep duration, sleep quality, and daytime sleepiness. The retrospective nature of the self-reported data may have resulted in underreporting. It is possible that the respondents tended to report shorter screening viewing times because they wanted to give a socially desirable answer. The underreporting may have weakened the association between screening time and sleep outcomes. On the other hand, the instruments used in this study may not be able to accurately account for the screening time from multiple screen use, as shown by the discrepancy between the total self-reported and total calculated screen viewing duration. Such a discrepancy may have resulted in over-reporting, as the screening time may be double when two devices are used at the same time. This over-reporting may also have resulted in the weakened association that was observed between screening time and sleep outcomes. Although it would be ideal to conduct field observations among the respondents in order to come to a more objective determination of screening time, the cost of doing so would be too high for this study given its large sample size (*N* = 762). On the other hand, the questionnaire used in this study did not cover all of the issues related to screen viewing. For example, the number of times the respondents viewed their devices over the past week, when the participants engaged in screen viewing, and the approach taken by parents on the use of digital devices. Finally, this study may not be generalizable to the adolescent population in Hong Kong, as the sample was selected from only one district.

## 6. Implications

Based on the information on current patterns of screen viewing and sleep among Hong Kong adolescents, health promotion programs can be established to address the potential health needs of local adolescents. This study also provides information for further studies on topics relating to the screen viewing and sleep habits of adolescents. Furthermore, as the association between portable video devices and sleep is a new finding, this provides a starting point for further investigations on the impact of portable video devices on adolescent health. In contrast, the absence of any association between television screening time and sleep outcomes suggests that the impact of television on adolescents may not be as strong nowadays as in the past. This also indicates that health promotion programs focusing on the use of television and personal computers could be shifted to target the use of mobile phones and portable video devices, because of advancements in technology.

## 7. Conclusions

Hong Kong adolescents getting inadequate sleep and experiencing occasional difficulties with sleeping, and spending more time viewing televisions, computers, mobile phones, and portable video devices. This study explored the impact of screen viewing on sleep duration, sleep quality, and daytime sleepiness among Hong Kong adolescents. Since negative effects were found between sleep duration, sleep quality, and daytime sleepiness and increased screen viewing of different types of devices, determining the recommended level of screen viewing among adolescents is a matter of public health. Moreover, tailor-made health education programs should be developed based on the results of this study to prevent adolescents from suffering from the negative effects induced by long screening times.
